# Supplementary material for: Ldha-Dependent Metabolic Programs in Sertoli Cells Regulate Spermiogenesis in Mouse Testis
Source: Biology (Basel). 2022 Dec 9;11(12):1791. doi: 10.3390/biology11121791 (PMC9775226; doi:10.3390/biology11121791)
Supplement: Supplementary file 1 [file biology-11-01791-s001.zip › supplement table S1 primer for geneotype and RT-PCR.pdf]

Primers for Genotyping		
LDHA- GenotypeF	CTGAGCACACCCATGTGAGA	WT: 458bp<br>Mutant: 600bp
LDHA- GenotypeR	AGCAACACTCCAAGTCAGGA	
MPC1- GenotypeF	TGTTTCCTTAGACAAAGTGTCCAG	WT: 450bp<br>Mutant:600
MPC1- GenotypeR	TCGGCTGTTTATTCAAGCTCC	
FrenchAmh-creGenotype1	CTATCGTGGATCAGCGACCTTC	WT: 100bp<br>Mutant: 180bp
FrenchAmh-	CACGGGAACGAAATGAACACC	
FrenchAmh-creGenotype3	GCGGTCTGGCAGTAAAAACTATC	
FrenchAmh-creGenotype4	GTGAAACAGCATTGCTGTCACTT	

Primers for Real-time quantitative		Product length
LDHA	AGGTCCCCAGAACAAGATT	134bp
	TCTCGCCCTTGAGTTTGTCT	
LDHB	AGGGAATGTACGGCATTGAG	105bp
	CCTCATCGTCCTTCAGCTTC	
LDHC	TTGGGACAAAGTGGTATC	294bp
	GTCTCAAAAAAAGGGGGA	
LDHD	TCCTGGTCGACCCAGATGAT	104bp
	TTCTCCAGTGCATGTCCCAC	
MPC1	GGTACAACCTCGAAACTGG	137bp
	TCAAGAGCTGGTCCTTGTACC	
MPC2	CCAAAGAAATTGAGGCCGCT	166bp
	ACCCTGTAGCCATCAACACAG	
