# Supplementary material for: Ldha-Dependent Metabolic Programs in Sertoli Cells Regulate Spermiogenesis in Mouse Testis
Source: Biology (Basel). 2022 Dec 9;11(12):1791. doi: 10.3390/biology11121791 (PMC9775226; doi:10.3390/biology11121791)
Supplement: Supplementary file 1 [file biology-11-01791-s001.zip › Supplement table S2 antibody and chemical.pdf]

Antibody	Manufacturer	Catalog No.
SOX9	Millipore	AB5535
LDHA	Abcam	Ab92903
GATA4	Santa Cruz	SC25310
PNA	Invitrogen™	L32458
Mitochondria	Molecular Probes	C1048
Donkey anti Rabbit 555	Abcam	Ab150074
Donkey anti Mouse 488	Proteintech	SA00013-5
Donkey anti Rabbit 488	Proteintech	SA00013-6

Chenical	Manufacturer	Catalog No.
IonomycinA23187	Sigma	56092-81-0
PMSG	Sudgen	72122
HCG	Sudgen	72120
HTF	Biorigin	BN80001
KSOM medium	ELITE-MEDIA	M3025-03
Choline	Sigma	C7519
