# Supplementary material for: Ldha-Dependent Metabolic Programs in Sertoli Cells Regulate Spermiogenesis in Mouse Testis
Source: Biology (Basel). 2022 Dec 9;11(12):1791. doi: 10.3390/biology11121791 (PMC9775226; doi:10.3390/biology11121791)
Supplement: Supplementary file 1 [file biology-11-01791-s001.zip › Supplemental Figures.pdf]

Supplemental Figure 1

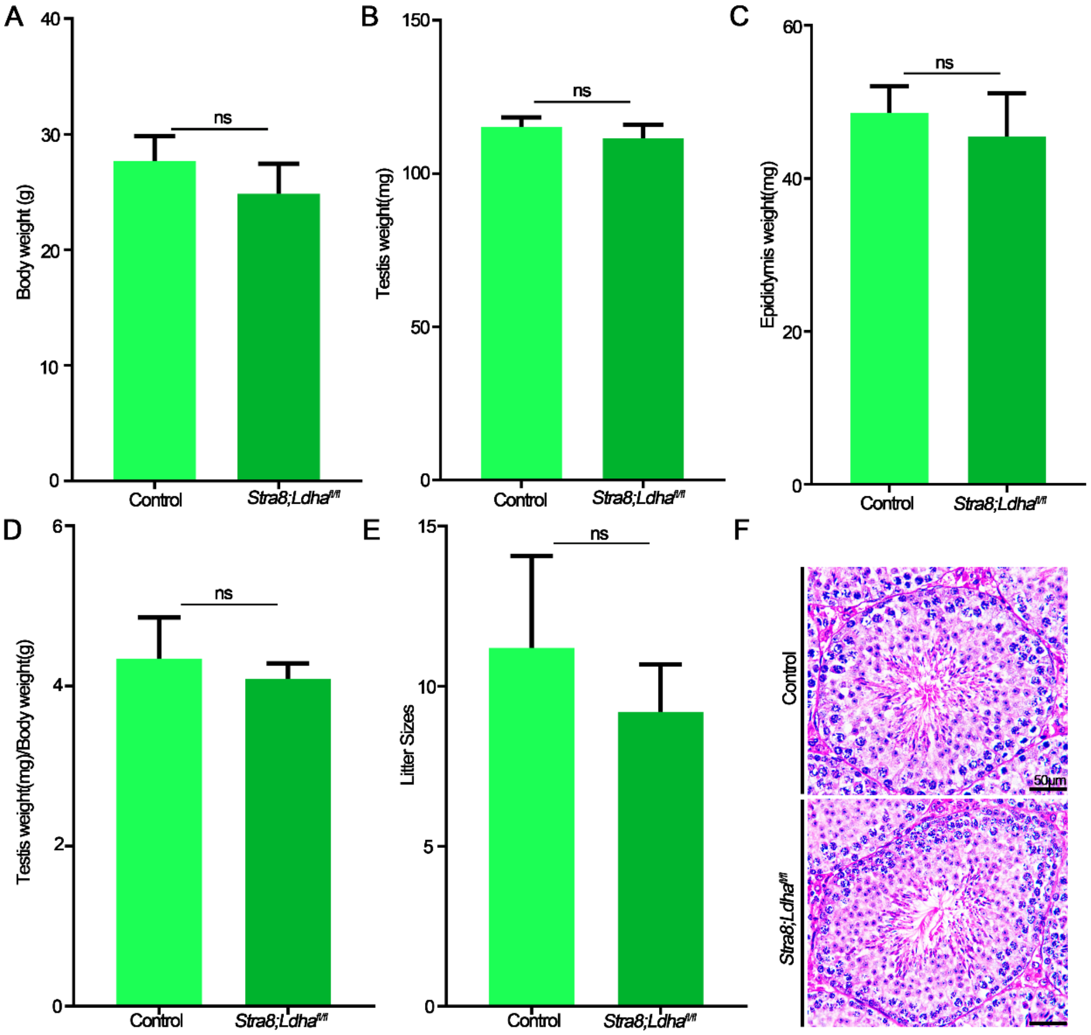

Supplemental Figure S1: Loss of *Ldha* in spermatogenic cells did not affect spermatogenesis. (A&B&C) Comparisons of body and testis and epididymis weight from male control and *Stra8-cre; Ldha$^{fl/fl}$* mice. ns indicates no difference. Data were analyzed using the mean ± SEM for three mice per genotype. (n=3) (D) Ratios of testes to body weight of three-month-old male control and *Stra8-cre; Ldha$^{fl/fl}$* mice (n=3). Data were analyzed using the mean ± SEM for three mice per genotype. ns indicates no difference. (E) Comparisons of litter size from three-month-old male control and *Stra8-cre; Ldha$^{fl/fl}$* mice (n=3). Data were analyzed using the mean ± SEM for three mice per genotype. ns indicates no difference. (F) Images of hematoxylin-eosin staining (H&E) of testes from three-month-old male control and *Stra8-cre;* Ldhafl/fl mice (scale bars, 50 μm).

Supplemental Figure 2

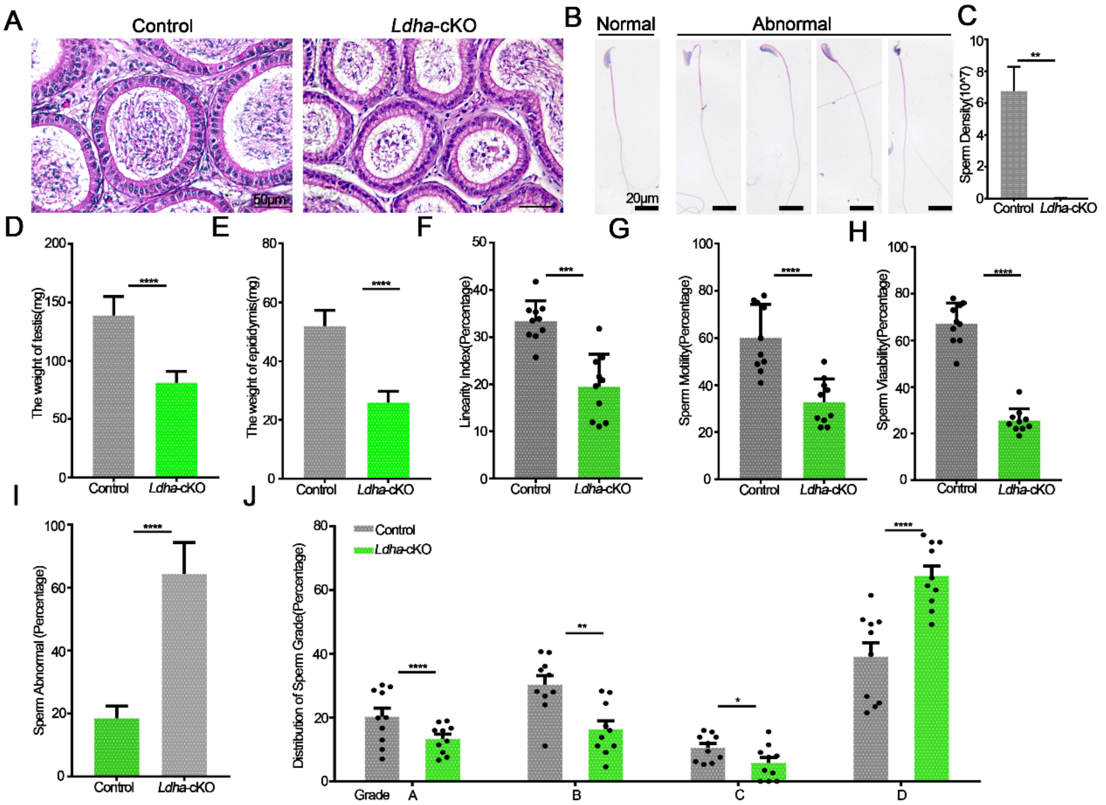

Supplemental Figure S2: Loss of *Ldha* in Sertoli cells resulted in reduced testis size and subfertility. (A) Representative images of H&E-stained cauda epididymides from three-month-old male control and *Ldha*-cKO mice. (Scale bar=50 μm). (B) Representative images of H&E-stained sperm from three-month-old male control and *Ldha*-cKO mice. Scale bar, 20 μm. (C) Comparisons of sperm density from male control and *Ldha*-cKO mice. ** indicates a significant difference of $P < 0.01$. Data were analyzed using the mean ± SEM for three mice per genotype. (n=3). (D&E) Comparisons of testis and epididymis from male control and *Ldha*-cKO mice. **** indicates a significant difference of $P < 0.0001$. Data were analyzed using the mean ± s.e.m. for three mice per genotype. (n=3) (F) Comparisons of the linearity index of sperm from male control (n=6) and *Ldha*-cKO mice (n=7). *** indicates a significant difference of $P < 0.001$. (G) Percentages of sperm motility from three-month-old male control (n=6) and *Ldha*-cKO mice (n=7). **** indicates a significant difference of $P < 0.0001$. (H) Percentages of sperm viability from three-month-old male control (n=6) and *Ldha*-cKO mice (n=7). **** indicates a significant difference of $P < 0.0001$. (I) Percentages of abnormal sperm from three-month-old male control (n=6) and *Ldha*-cKO mice (n=7). **** indicates a significant difference of $P < 0.0001$. (J) Percentages of grade sperm from three-month-old male control (n=6) and *Ldha*-cKO mice (n=7). ****, **and * indicate a significant difference of $p < 0.0001$, 0.01 and 0.05, respectively.

Supplemental Figure 3

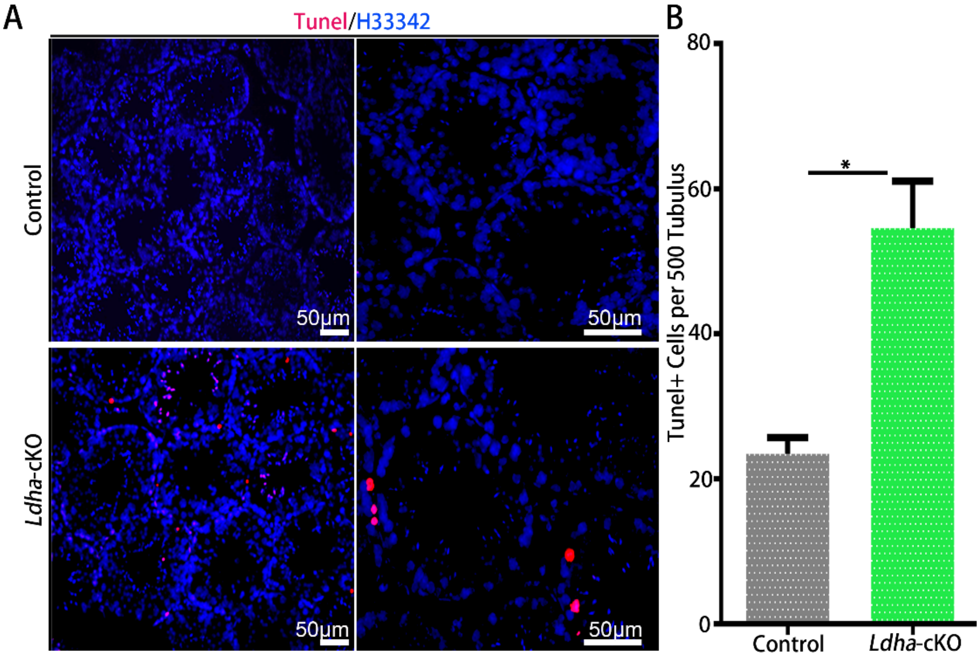

Supplemental Figure S3: Loss of *Ldha* in Sertoli cells resulted in increased apoptosis in the testis. (A) Immunofluorescence staining of TUNEL in cross-sections of testes from control and *Ldha*-cKO males at 2 months. (scale bar =50 μm). (B) Quantification of $^{TUNEL+}$ cells in the testes of control and *Ldha*-cKO males. (n=3). * indicates a significant difference at P<0.05.

Supplemental Figure 4

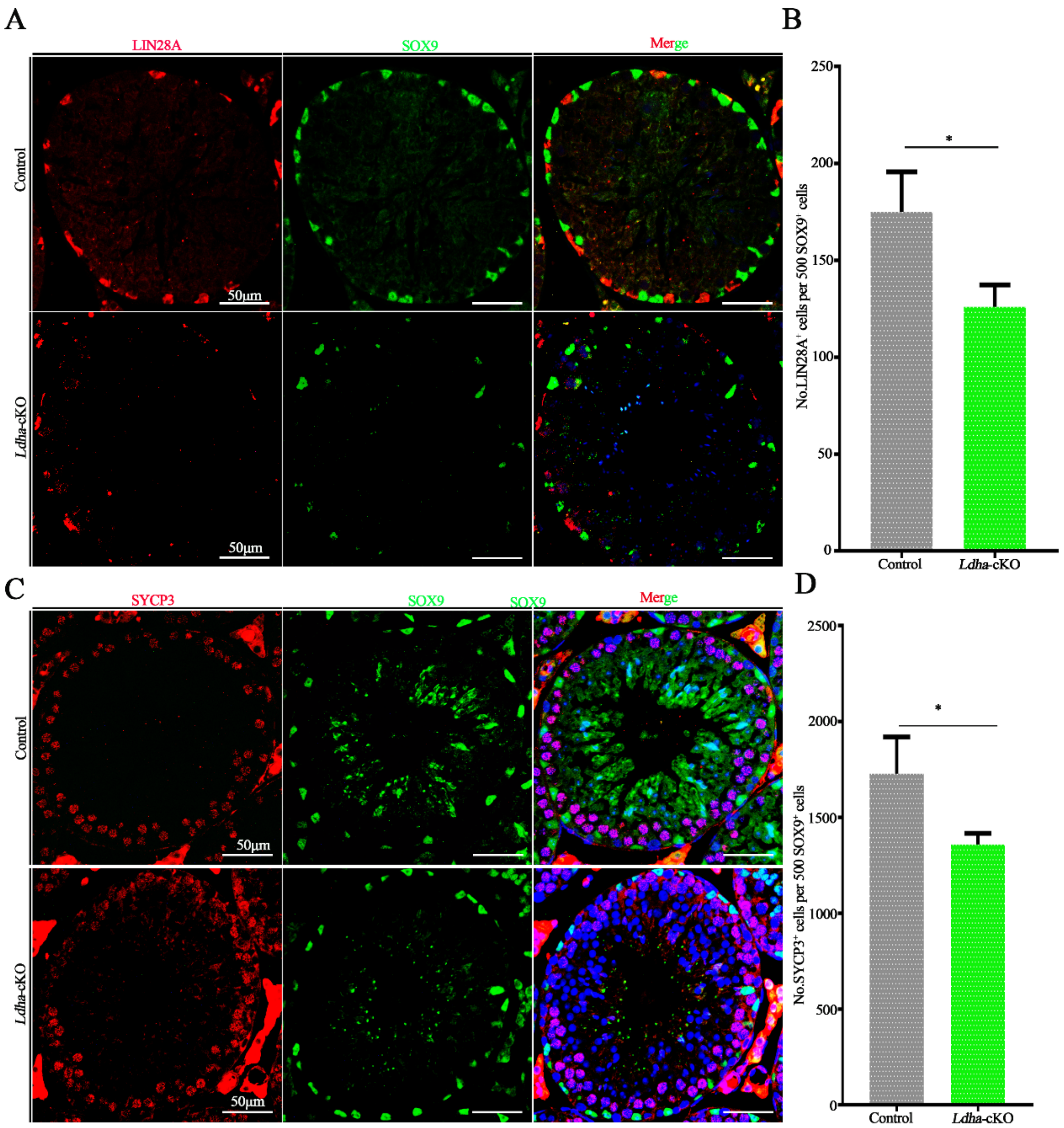

Supplemental Figure S4: Loss of *Ldha* in Sertoli cells affected spermatogonia and spermatocyte cells. (A) Immunofluorescence staining for SOX9 and LIN28A in cross-sections of testes from control and *Ldha*-cKO male mice. (scale bar =50 μm). (B) Quantification of spermatogonia per 500 Sertoli cells in the testes from control and *Ldha*-cKO male mice. (n=3). * indicates a significant difference of $P < 0.05$. (C) Immunofluorescence staining for SOX9 and SYCP3 in cross-sections of testes from control and *Ldha*-cKO male mice. (scale bar =50 μm). (D) Quantification of spermatocytes per 500 Sertoli cells in the testes from control and *Ldha*-cKO male mice. n=3. * indicates a significant difference of $P < 0.05$.

Supplemental Figure 5

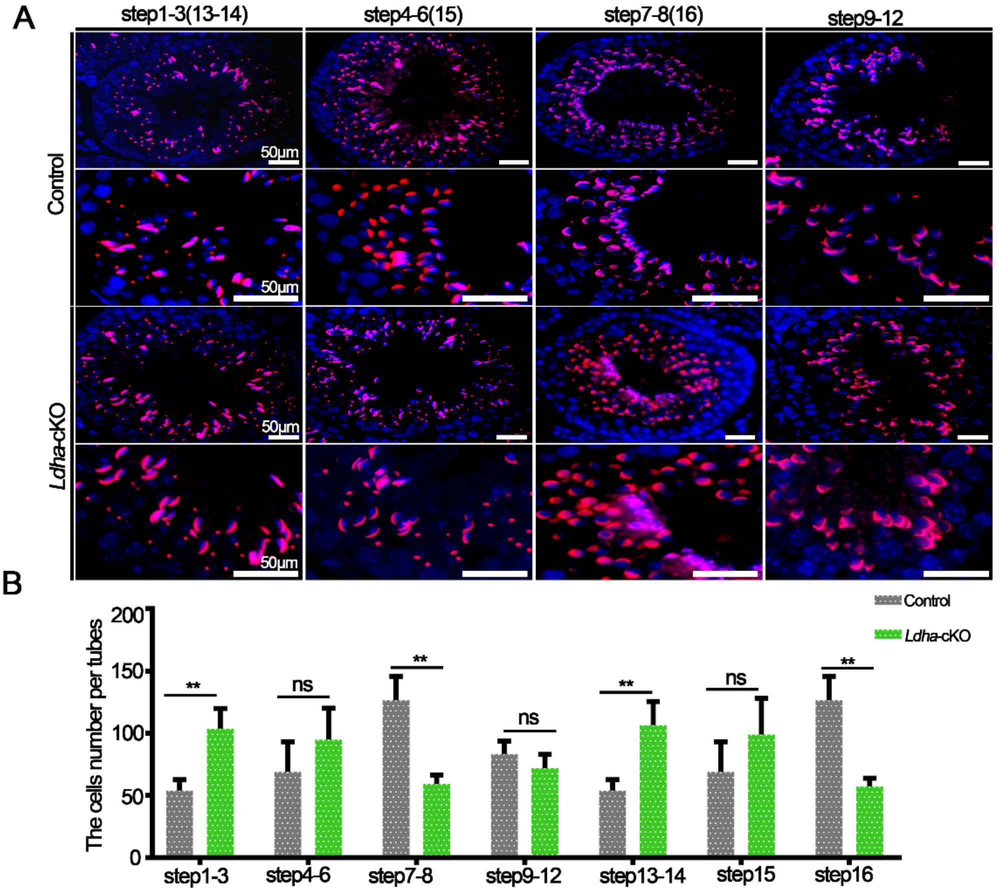

Supplemental Figure S5: Loss of *Ldha* in Sertoli cells caused defects in spermiogenesis. (A) Immunofluorescence staining for PNA in cross-sections of testes from male control and *Ldha*-cKO mice. (scale bar =50 μm). (B) Quantification of the number of different stages spermatozoa of meiosis per mouse of control and *Ldha*-cKO male mice. n=3. ** indicates a significant difference of $P < 0.01$. ns indicates no difference.

Supplemental Figure 6

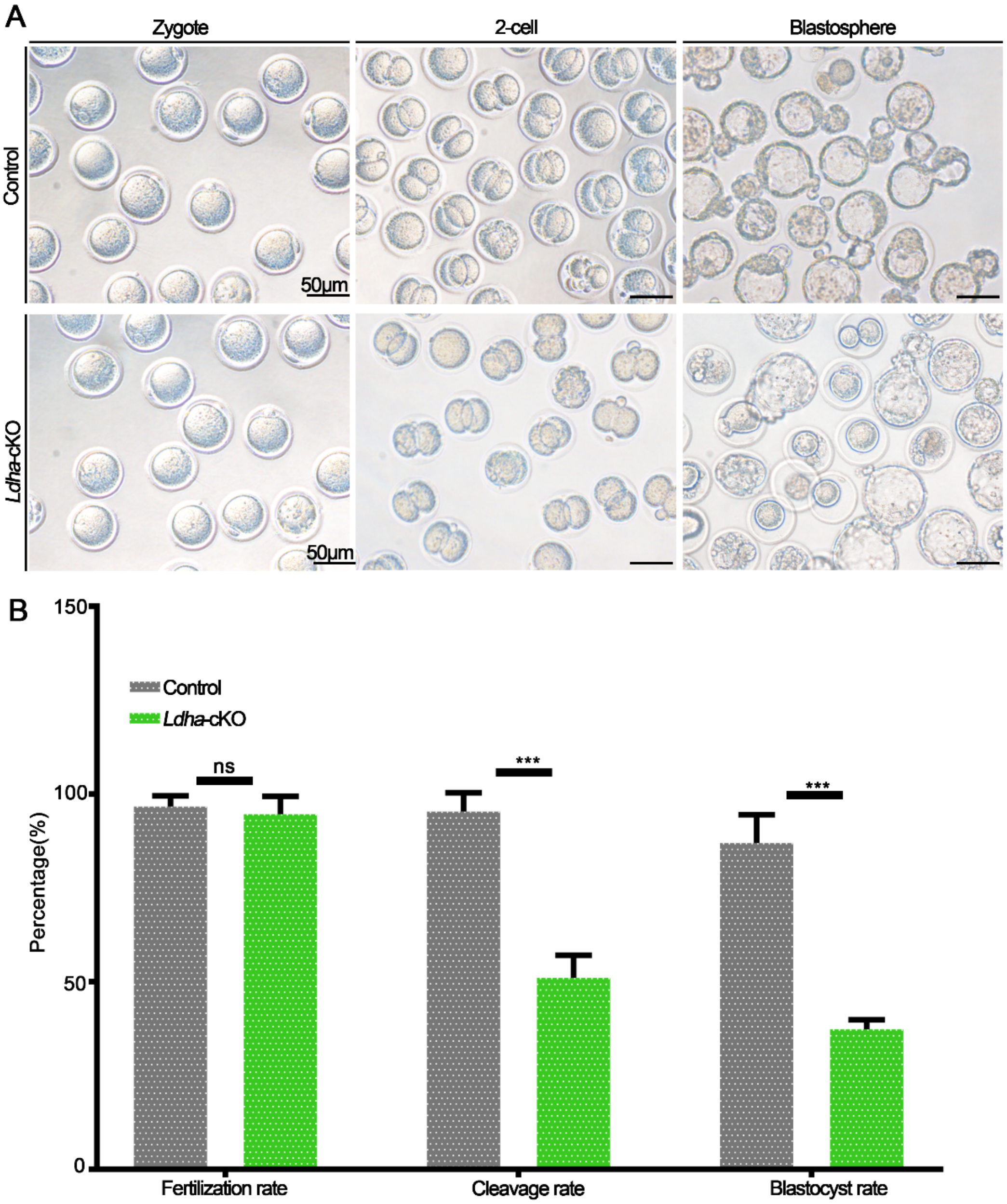

Supplemental Figure S6: Fertilization capacity of sperm from *Ldha*-cKO animals was affected. (A) Representative images of IVF outcomes from male control and *Ldha*-cKO mouse sperm (n =3). (scale bars= 50 μm). (B) Fertilization, cleavage and blastocyst rates from male control and *Ldha*-cKO mice. At least 100 oocytes were used per group. *** indicates a significant difference of $P < 0.001$. ns indicates no difference.

Supplemental Figure 7

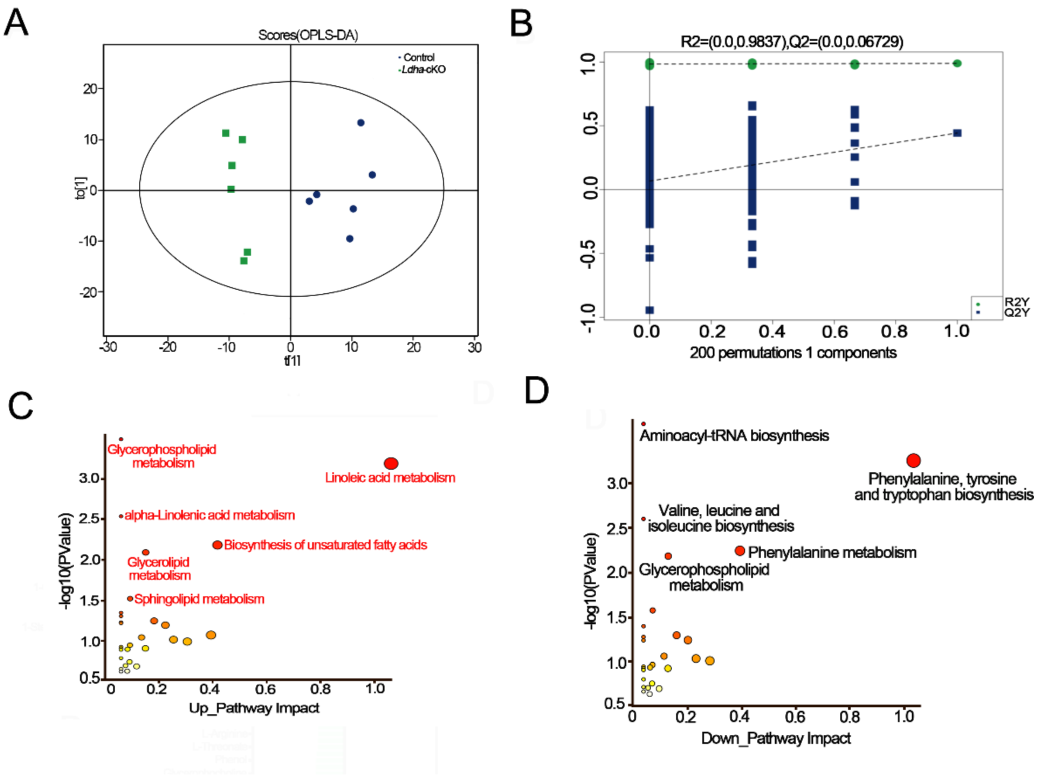

Supplemental Figure S7: Quality control and metabolomic analysis of Ldha loss in Sertoli cells. (A) A score plot showing group separation in an OPLS-DA score space. (B) The response permutation test plot (n = 200) for the OPLS-DA model in (A). The R2 and Q2 values of the permutated model are represented on the head of the plot, corresponding to y-axis intercepts: R2 = (0.0, 0.9837) and Q2 = (0.0, 0.06729). (C-D) Overview of up- and downmetabolites of sperm in male control (n=6) and Ldha-cKO mice (n=7).

Supplemental Figure 8

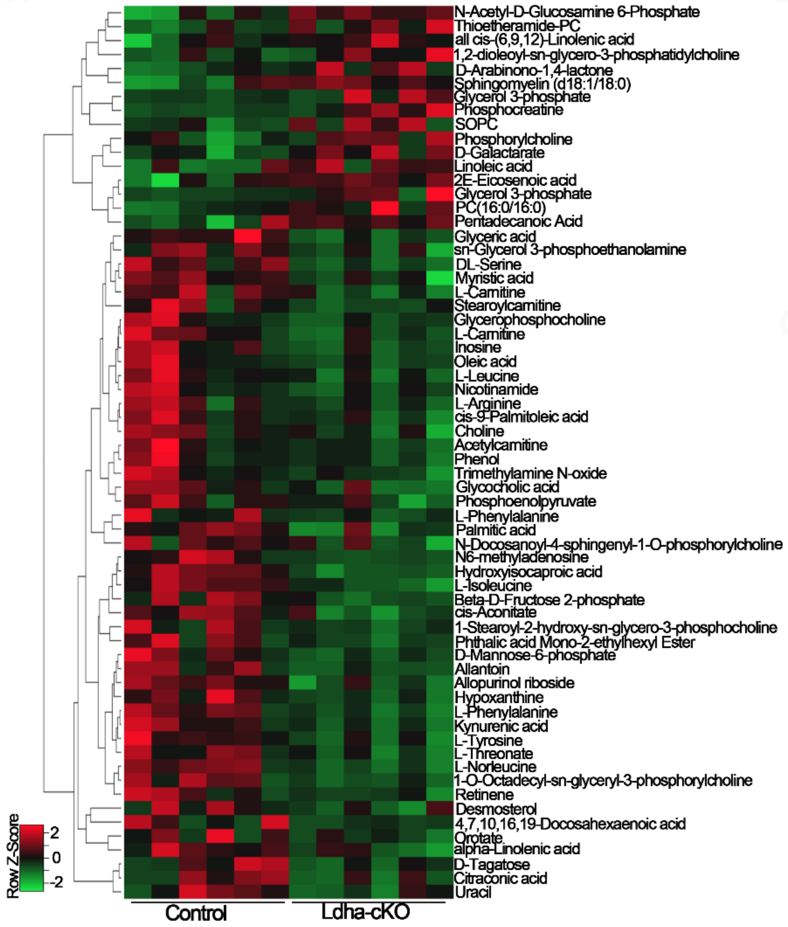

Supplemental Figure S8: Heatmap of metabolite profiles identified in sperm samples from control and *Ldha*-cKO mice.

Supplemental Figure 9

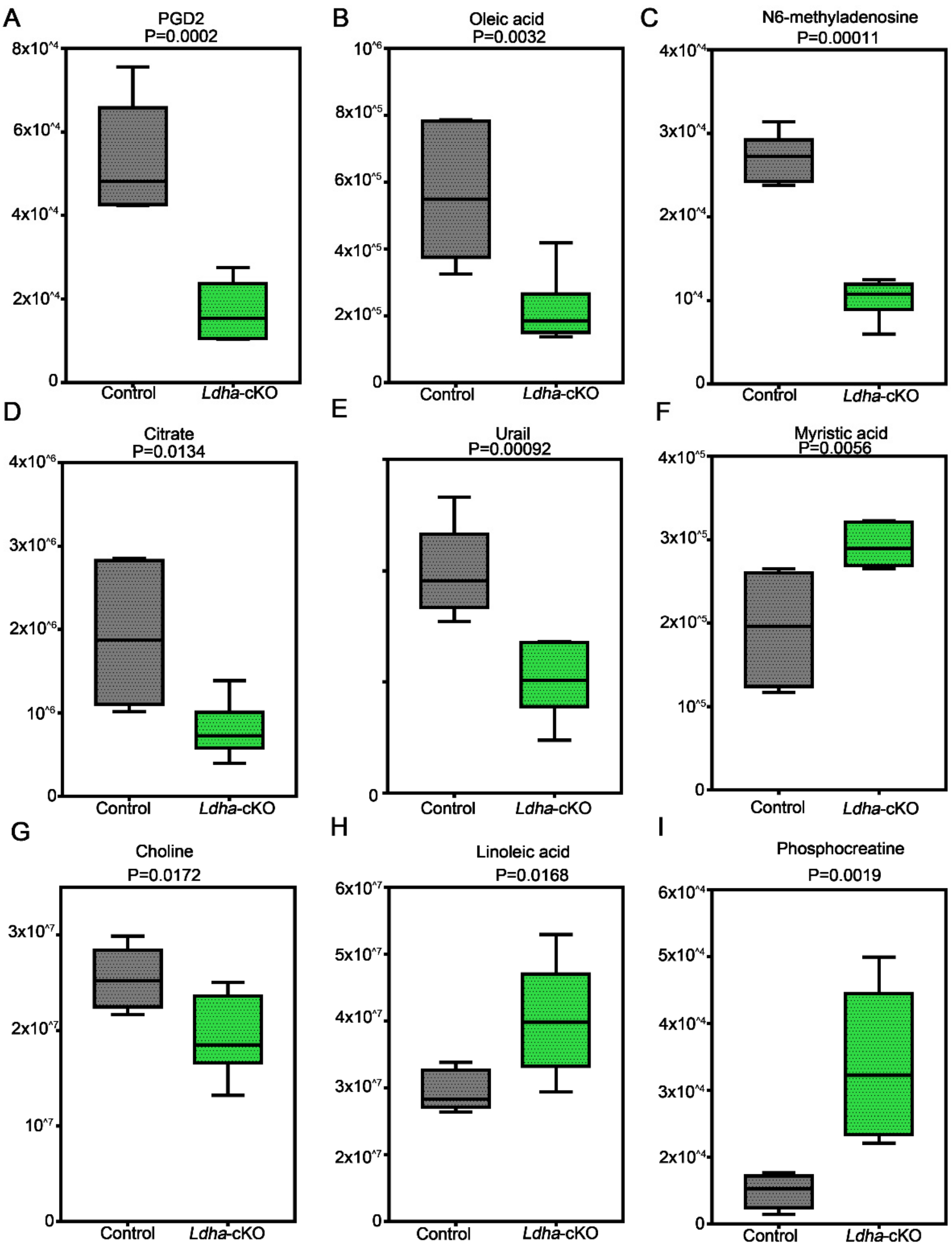

Supplemental Figure S9 Metabolomic dysregulations in sperm from *Ldha*-cKO compared to control male mice. (A-I) Box plots showing representative metabolite changes in sperm between control and *Ldha*-cKO mice.

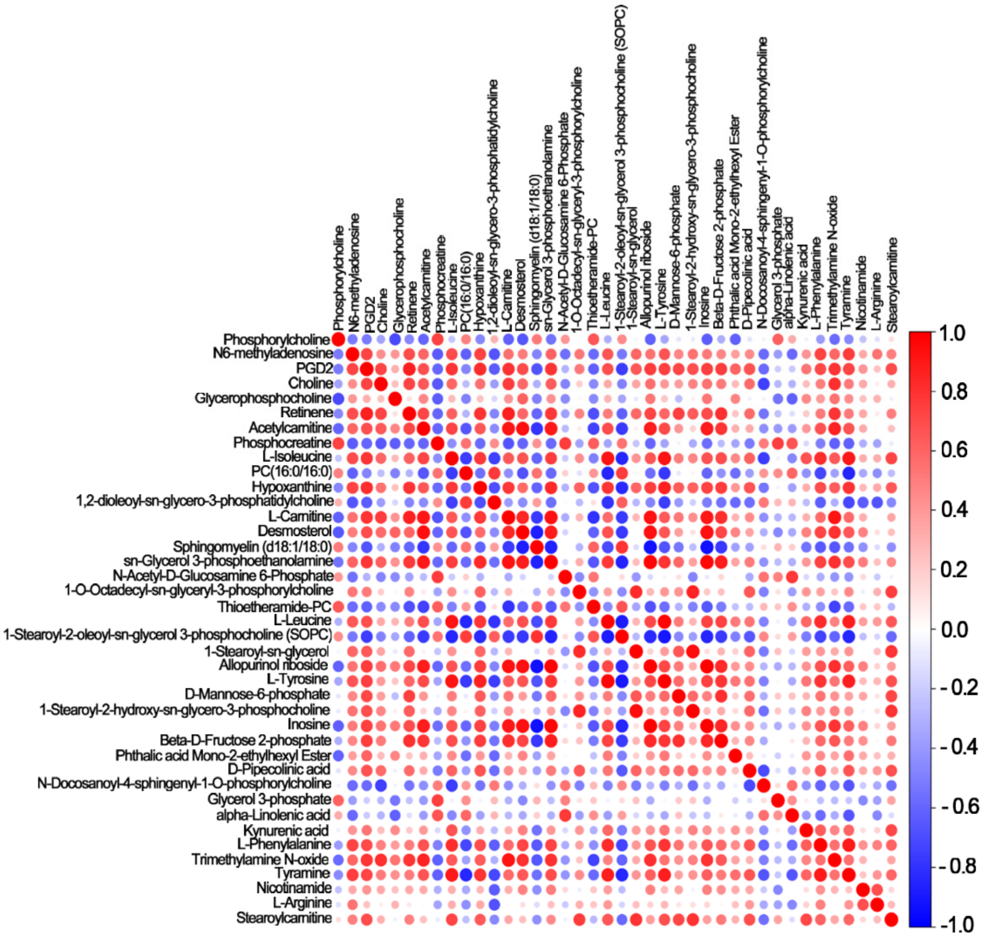

Supplemental Figure S10: Differential metabolite correlation heatmap. Differential metabolite association analysis was conducted to examine the consistency of the trend of metabolites and metabolites and to analyze the correlation between each metabolite in sperm of the *Ldha*-cKO (n=7) compared to controls (n=6) by calculating the Pearson correlation coefficient (shown in vertical coordinates) between all the two metabolites. The correlation of metabolites revealed the synergy of changes among metabolites. Color represents correlation, red was positively correlated, blue was negatively correlated, and the darker the color, the greater the correlation.

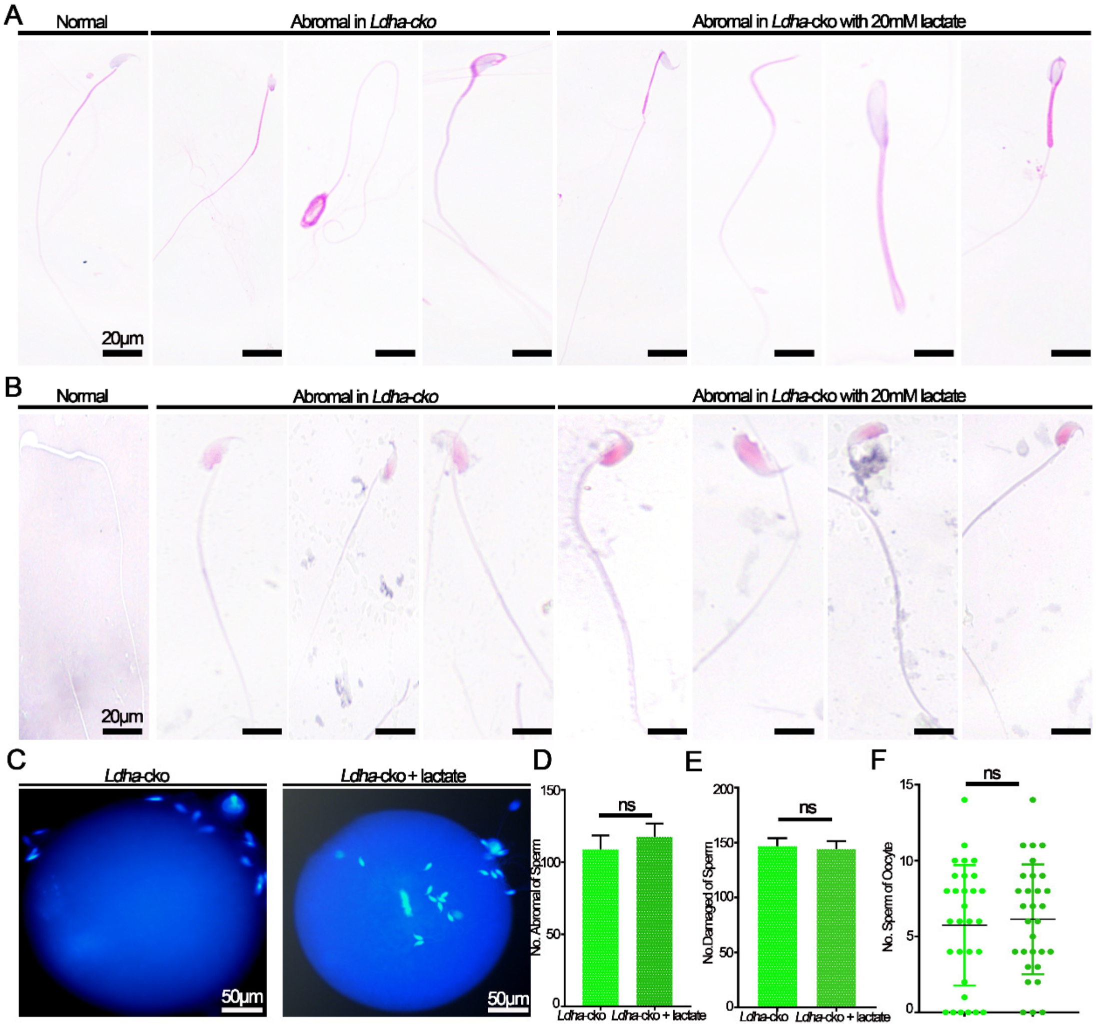

Supplemental Figure S11: Lactate supplementation did not rescue defects in sperm function. (A) Image and quantification (D) of abnormal sperm in *Ldha*-cKO mouse sperm supplemented with 20 mM lactate or dimethyl sulfoxide control. (scale bar =20 μm) (n=4). (B) Image and quantification (E) of damaged sperm in the *Ldha*-cKO mice treated with 20 mM lactate for 60 min and *Ldha*-cKO mice treated with dimethyl sulfoxide for 60 min. (scale bar =20 μm) (n=4). (C&F) Image and quantification (D) of sperm adhered to oocytes in *Ldha*-cKO mouse sperm treated with 20 mM lactate and *Ldha*-cKO mouse sperm treated with dimethyl sulfoxide as controls (scale bar =50 μm). Fifty oocytes were counted in the test. Ns indicates that there is no difference. In each mouse, 200 sperms were counted and
used to calculate the total percentage of abnormal sperm morphology.
